# Intervening on Burnout in Complex Organizations – The Incomplete Process of an Action Research in the Hospital

**DOI:** 10.3389/fpsyg.2020.02203

**Published:** 2020-09-23

**Authors:** Sara Ramos, Patrícia Costa, Ana M. Passos, Sílvia A. Silva, Ema Sacadura-Leite

**Affiliations:** ^1^DINÂMIA’CET-IUL, ISCTE-IUL, Lisbon, Portugal; ^2^Business Research Unit (BRU-IUL), ISCTE-IUL, Lisbon, Portugal; ^3^Occupational Health Service, North Lisbon University Hospital Center, Lisbon, Portugal; ^4^NOVA National School of Public Health, Public Health Research Centre, Universidade NOVA de Lisboa, Lisbon, Portugal; ^5^Faculdade de Medicina, Universidade de Lisboa, Lisbon, Portugal

**Keywords:** action research, burnout, health professionals, intervention, leadership

## Abstract

Health professionals are at high risk for developing burnout symptoms. Directed at reducing the organizational variables affecting professionals’ burnout, an action research was developed in a specific sector of a large hospital, with 59 doctors, 66 nurses, and 42 ancilliary professionals. Researchers conducted 11 interviews, one focus group, and 20 h of *in loco* observation. Professionals report demotivation and the need to address the emotional part of their job. Nonetheless, the hierarchy blocked the proposed intervention possibilities. Organizational factors are unequivocally relevant, particularly in complex settings with emotionally charged interactions, and the direct hierarchy is pivotal for facilitating organizational change.

## Introduction

Hospitals are stressful working places. Emergency rooms are usually filled with many patients with distinct needs; each specific service needs to attend to its own inpatient demands, as well as other services’ eventual necessities; some decisions need to be made and acted upon quickly; doctors, nurses, and ancillary professionals struggle to coordinate their complementary tasks in a timely manner; families await to see their loved ones and grieve their losses; good news and bad news fill the corridors and hope or despair fill the waiting rooms or the nurseries. Health professionals have, therefore, demanding jobs in many areas, including cognition, information process and decision making, emotion and affect management, interpersonal tensions, or administrative burdens (e.g., Ghodse and Galea, 2006).

Not surprisingly, the prevalence of stress is high among health professionals. On one of its extreme manifestations, burnout, the prevalence among doctors is as high as 75% (Clough et al., 2017), with high numbers reported both in the United States and in Europe (Soler et al., 2008; Aiken et al., 2012). As a consequence of high levels of stress among health professionals, two major interrelated concerns arise. First, the impairment of the health and well-being of those professionals due to occupational stress. Second, the consequences for the quality of care provided to the patients, with burnout as a strong predictor of self-reported medical errors (e.g., Hall et al., 2016). Hence, diverse interventions to reduce burnout and occupational stress have been proposed and implemented over the last decades (for a systematic review, please refer to Clough et al., 2017).

The present paper describes a specific action research case study, in an inpatient clinical department at a major European Hospital. The main goals of the paper are not only to describe a detailed intervention with a particular methodology but also to highlight the main obstacles faced by the research team in this process. As a result, we hope to contribute to the reflection about the challenges of implementing change in the context of health services.

The intervention we describe was embedded in a larger European project, developed over a 5 year period. The main goals of the project were to monitor the burnout levels of health professionals, to identify the key organizational culture factors related to burnout and the consequent decrease in the quality of care provided to the patients. The last phase’s main goal was to develop bottom-up solutions and interventions to address those issues and improve professionals’ health and patient safety, within an action research paradigm.

According to a systematic review by Clough et al. (2017), the most common interventions in workplace stress and burnout within the context of health services tends to focus on organizational-level changes such as reducing working hours or caseloads, with mixed outcomes. At the individual level, interventions based on cognitive-behavioral, relaxation, and supportive discussion strategies tend to dominate the field.

In recent years, occupational health interventions targeting the organizational level (in comparison with an individual level intervention) have been recommended as an important strategy for stress and burnout prevention. These interventions frequently focus the causes of job stress (stressors) and aim at improving the psychosocial work conditions and employee health and well-being (Nielsen et al., 2010). Simultaneously, it is recognized that such interventions are complex and many factors may influence whether they succeed or not (e.g., Nielsen et al., 2010). It appears that these interventions may be more successful if a participatory intervention process is used. Nielsen et al. (2010) identify three main reasons for sustaining the participation positive effects. Firstly, participation allows a customization of the intervention to the organizational context and culture because it allows to consider the employees’ job expertise and knowledge of the organizational context. Secondly, studies have suggested participatory approach as one important working mechanism that explained the effects of an organizational intervention (e.g., Nielsen et al., 2010). Essentially because this approach gives an important role to employees in an empowerment process and it is likely to increase worker perceived responsibility, control, sense of fairness, and justice. Thirdly, participation may contribute to facilitate the change process and decrease some resistances to change.

Within the literature on stress-related interventions, several authors (e.g., Vignoli et al., 2017) emphasize the need to understand and consider the context in order to better diagnose and plan an occupational health intervention. Considering the Hospital context, some of the most commonly cited stressors include dealing with the death of patients and their families, long working hours, responsibilities of the role, aggression toward the professionals, time pressure, work shifts, and conflict among coworkers (e.g., Isikhan et al., 2004; Winstanley and Whittington, 2004; Huntington et al., 2008; Golubric et al., 2009). Despite these common stressors, it is only possible to identify specific job stressors if we use an open approach of data collection sensitive to the reality of the group or organization. Using a qualitative approach (as for instance the action research) is an important strategy for acknowledging the importance of the local context and to adjust the intervention accordingly.

Action research (AR) has already a long history in the social and human sciences. It is an approach that combines action with reflection, theory with practice, and that involves organizational actors in the research process itself (Bradbury, 2015). In 1946, Kurt Lewin published the first article in which he makes an explicit reference to action-research. According to the author, AR is an especially effective methodology in solving complex social problems and promoting change both in terms of individual behaviors and of organizational dynamics. After more than eight decades since this publication, AR has been used to study and to make interventions in several areas such as education (Tuncel, 2017), medicine (McCabe et al., 2014), and information technology (Madden et al., 2014).

AR can be defined as a cyclical process that allows “learn by doing.” In this process, researcher and practitioners collaborate along the different stages to identify relevant problems or issues, to plan and implement actions, and to evaluate and reflect about the process and outcomes (French and Bell, 1995; Montgomery et al., 2015). In the health context, action-research has been used to develop practical solutions to real problems while allowing those involved in the process to develop their understanding and knowledge of the situation and also to systematically identify issues and problems to improve (Montgomery et al., 2015), therefore, fitting the recommendations for success of interventions in the health context. In fact, we find in the literature several studies that have used action-research to promote the quality of care (e.g., Nolan and Grant, 1993) and new organizational models (e.g., Dickinson et al., 2005) or to reduce psychosocial risks of health professionals (e.g., Montgomery et al., 2015). AR advocates a bottom-up approach to problem solving, through which health professionals, in collaboration with researchers, identify relevant problems and/or the needs for change in their context and, together, develop, implement, and evaluate specific solutions. However, most interventions to reduce psychosocial risks of health professionals, such as burnout, focus essentially on the individual, ignoring the influence of organizational context or even the work teams in which health professionals carry out their activity (Pisljar et al., 2011). As Montgomery (2014) points out, it is necessary to develop an approach centered on the organizational system to promote change and develop healthier work environments.

To fill this gap, the European Project ORCαB – *Improving quality and safety in the hospital: the link between organizational culture, burnout and quality of care* – proposed a specific AR model for the health context which comprises five cyclical stages: (1) problem identification, (2) action planning, (3) implementation, (4) evaluation, and (5) reflection. The duration of each stage can vary considerably from situation to situation or there may even be some overlap between the stages.

### Stage 1: Problem Identification

The first stage of AR consists in identifying and selecting a problem. It is essentially a diagnostic phase that allows the identification of problems that require greater attention from the point of view of the stakeholders. In addition to the direct involvement of top management, it is also essential to involve health professionals in defining the problem, using different methods and techniques of data collection, such as observation, individual and group interviews, and document analysis. This first stage ends with the identification of the problem and possible solutions by the health professionals involved.

### Stage 2: Action Planning

The main goal in this stage is to critically assess the feasibility of possible solutions, giving special attention both to the resources (human and technological) need for implementation and to the internal alliances necessary to develop them. Participants should be invited to analyze the identified problems in greater detail and to think about possible solutions, namely through brainstorming sessions. The direct involvement of health professionals is a fundamental aspect that increases the likelihood of success. This step should end with the identification of a solution to the problem and conducting a pilot study to test the feasibility of that solution.

### Stage 3: Implementation

This stage consists of the implementation of solutions to the problems identified in the initial two stages. Although the pilot study aims to ensure the viability of the solutions, it is important that the team adopts a step-by-step approach in its implementation, i.e., start with a possible solution and gradually adopt new solutions as soon as the first has been accepted. In the health context, for the success of this step, it is important to promote workshops and training sessions to improve the knowledge and confidence of health professionals in several domains. Moreover, AR team should be open to new solutions that can emerge during the implementation of a specific solution or to the redefinition of solution itself.

### Stage 4: Evaluation

This stage requires the use of research methods and rigorous criteria to assess the extent to which AR had the expected outcomes (e.g., satisfaction levels, absenteeism, professional burnout levels, and medical errors) but also the way in which the process was carried out.

### Stage 5: Reflection

The last stage allows the team and the health professionals involved in the AR to be more aware of both the process and its results, the situation, and the context in general. To assess the quality of the AR, the team and the health professionals involved should reflect on whether participation was effectively achieved, whether the team was able to involve professionals with different perspectives on potential problems and solutions, and whether they were able to focus on significant issues.

These stages should work as guidelines to develop an AR project in the health context. It is mandatory that the team responsible for the AR project understands the specific nature of these services and departments and that the project does not compromise its functioning. Moreover, although the success of an action-research project depends on the collaboration of health professionals, it is important to assure that they are not unduly loaded with additional tasks.

To sum up, action research (AR) differs from traditional empirical research in the sense that its focus on researching *with* participants and not *about* participants, with the acquisition of knowledge from both researchers and participants as one of its key goal (Montgomery et al, 2015).

## Materials and Methods

### Participants and Context

The intervention was carried out at a major teaching hospital in a European capital, with the active participation of its Occupational Health Department. This is a large central hospital. Considering the dimension of the hospital and after discussing with the Occupational Health Department director, an inpatient clinical department was chosen as the target population for the action research. This particular service was chosen for three main sets of reasons. First, it has one of the highest occupancy rate of the hospital and its inpatients are mostly elderly with multiple pathologies, and a high mortality rate, which creates a demanding environment for health professionals, therefore, putting them at a higher risk for stress-related issues. Some of the patients, because of their age and/or severity of their condition, have to stay at the hospital for repeated periods of time, which leads to the development of stronger ties between the patients and their families and the health professionals, which subsequently leads to a greater emotional weight of negative outcomes. Second, the specific sector chosen was also mentioned by the Chief Nurse of the Hospital as a service with a history of interpersonal friction among its professionals, hence reflecting yet another risk factor for burnout. Third, because of the multiple pathologies of its inpatients, this is a service which needs to be in permanent contact with other specialties within the Hospital. This creates interdependencies and competition for limited resources (professionals, equipment, and diagnostic resources), which many times results in increased conflict levels between this service and others.

The inpatient clinical department has several sectors, all coordinated by a Senior Specialist that is also Full Professor at the University. Within the department, there are overall more than 100 of workers including doctors, nurses, ancilliary personnel, administrative assistants, a psychologist, social assistants, and a nutritional technician. Overall, at the time of the intervention, the department had 81 beds, an average of 84 patients per day, and an average occupancy rate of 97.3%. In average, the internment of the patients’ lasted for 6.6 days and the department reported a high mortality rate of 8.5%. Our intervention was developed in one of those several sectors (sector A).

Sector A has 21 beds and is divided in four teams, each coordinated by a senior specialist, responsible for some younger residents. With an occupancy rate averaging over 100%, it is usual to have patients stay in stretchers in the corridor, due to a lack of available beds, up to a maximum of 10 stretchers. Overall, the sector comprises 13 doctors (including the head of the sector, doctors, and residents), 17 nurses, and 10 health assistants. Being a teaching hospital with a strong link to the university, the focus of physicians is simultaneously on providing care for the patients but also on training younger professionals and developing research. Each of the four teams was also composed of nurses, supervised by the Chief Nurse. The nurses from this sector were mostly young and the group presented a high turnover rate.

### Action Research Process

#### Phase 1: Problem Identification

The first phase of the AR (problem identification) had two major goals: (1) to gather the maximum amount of information possible from the health professionals about what they considered as the biggest obstacles to their performance and well-being at work and, simultaneously, (2) to engage the professionals in the AR, expecting to increase their cooperation in subsequent stages of the project that is, by definition, necessarily collaborative, in line with the recommendations for a participative diagnostic as leverage of success.

For the project’s kick off, the team had a first meeting with the Director of inpatient clinical department, for a formal presentation of the project and a first overview of the service. Then, a second meeting was held with the head of the specific sector we would focus on and the respective head of nurses, who confirmed their interest in working with the team. The goal of the meeting was to make a first diagnosis of the service and to define the calendar for the action research, as well as to define a dissemination strategy to keep all professionals engaged and informed about what was going to happen at all phases. To disseminate the project internally, the team created a name for the project (SaudAR – a work that means “to greet” in the local language, and that is written mixing the word “saúde” (health) and the suffix “AR,” here reflecting action research). The team also distributed a flyer to all health professionals of the service, and emailed all the professionals the link of a blog where everyone from the service could post messages, give opinions, or ask questions at any point of the project. The head of nurses formally introduced the intervention team to all doctors, residents, medicine students, and nurses and we had the opportunity to answer all the questions about the project.

##### Data Collection

Data gathering was made using different qualitative methodologies, to address the need for triangulation by using multiple sources of evidence (Yin, 2018).

Two members of the team performed 20 h of direct unstructured observation, in the role of total observer (Waddington, 2004). They spent those hours at the sector at different week days and diverse times of the day, to have a complete picture of the rhythm and pace of the work, the movement of people during the day, and the type of dynamics and interactions between professionals, teams, patients, and patient families. Because the goal was to gather as much information as possible and considering that there were no previous hypotheses, the researchers did not develop a structured observation grid but rather toke notes of every event that was relevant. The observers registered key words and phrases, as well as who had said them, took notes about the sequence of events and took some time to write full notes immediately after leaving the location. Initially, the two researchers went together twice to the observation setting, to allow them a brief training period. After the observation moments, they met to discuss their individual notes and compare and contrast the aspects they had observed. In the subsequent observation moments, each researcher went alone. During these moments of observation, we had the chance to be present during two shift changes with the nurses’ team, as well as to accompany the doctors’ morning visit to their inpatients, where they would not only check on the patient but also take the opportunity to present the case to their medicine students.

The team interviewed 12 workers individually, selected based on diversity criteria (six doctors, five nurses, and one psychologist), to collect as many different viewpoints as possible (Bauer and Gaskell, 2000) and conducted a focus group with three ancilliary professionals, always using the same script of questions. The interview was semi structured (Flick, 2018), allowing for comparing between participants, but also to grant researchers the necessary freedom to explore some issues that might arise during each interview in more depth. Participants were asked to describe the sector (its key characteristics and a normal day at the service), to point out what they consider to be critical aspects of the sector that negatively impact their performance and well-being, as well as the aspects that are considered as adding value to the sector, to describe a situation that clearly reflect both, and finally to highlight three aspects that are considered as most important to be changed. The interviews lasted, in average, 20 min, and were conducted at the services’ facilities, during professionals’ breaks. To ensure privacy, all interviews were conducted in rooms with closed doors, and confidentiality was guaranteed to all the participants.

##### Data Analysis

The combined data from observation and research were analyzed by three researchers in a collaborative process (Clarke and Braun, 2013). We have employed thematic analysis to identify, analyze, and interpret patterns of meaning within our data (Braun and Clarke, 2006). More specifically, and considering the existence of multiple coders, we opted for template analysis (King and Brooks, 2016) to organize data. As the whole AR process is inductive by nature, categories were not determined *a priori*. Rather, an open approach was adopted, in which all themes were identified a posteriori, as a result of analyzing the data (Krippendorff, 2004/2018). During this process, we assigned one category to each relevant segment of the interviews and of the observation notes. If the segment’s content was not covered under the existing categories, a new category was created.

In the following section, we present the data globally, as an organized description of the most relevant data collected through the different means, and that informed the intervention proposal.

## Results

During data collection, health professionals were collaborative and supportive of the process, actively participating in the interviews and facilitating the observation process. The blog was, unfortunately, not used by the professionals, regardless of the several questions for discussion that the team included regularly. The main challenges faced by the research team were the lack of availability of some professionals in specific moments, due to the high occupancy rate of the sector.

The professionals describe the sector as technically excellent. In general, interpersonal relationships within professional groups are good, particularly within the nurses and young professionals (mostly residents and medical students). One of the most positive aspects of the sector is its potential in terms of scientific production (patients with complex situations and multiple pathologies) and teaching. However, data analysis also allowed to identify some issues considered as obstacles to effective functioning by the professionals.

At a macro/organizational level, the occupancy rate is a clear problem. On the one hand, the existence of a great number of stretchers in the corridors is mentioned by the majority of professionals as a main obstacle: “*Stretchers also have an impact, it limits our observation, there is no privacy, no intimacy to ask certain questions” (Doctor);* “*To give a bath in a stretcher is chaotic, especially with patients with a reduced mobility, or obese…” (Nurse)*. On the other hand, when the occupancy rate is over 100%, there is an insufficient nurses/ancilliary personal ratio. Professionals consider that “*teams are dimensioned to a certain number of patients; stretchers are a supplementary effort; we once had 19 stretchers, when there was no limit*” (Nurse). Adding to this problem, data from both the interviews and the observations highlighted very bustling sector. The corridors are often filled with people, mostly students, and residents that follow a senior doctor during his visits to patients but also relatives and visitors: “*Sometimes we need to ask students to leave … when there is a critical situation and we have 30 people wandering around” (Chief Nurse)*. Finally, doctors tend to consider that the informatics system is inadequate for their needs, because it takes a long time for the software to run, and that limits their capacity for providing care in a timely manner.

In what the specific sector is concerned, we have observed that most of the interactions occur between nurses and doctors. The ancilliary personnel seem inexistent, other professionals never mention them during the interviews, and this is reflected on their complaints: “*they could say ‘you are a good team, you did well today’, but they do not. No matter how hard we work, they never say thank you*.” Another obstacle for delivering good quality of care is a lack of information sharing. This happens between services (“*There are other specialties that do every kind of boycott to the Service*”), and within the professionals of the sector [“*There are no formal meetings between the medical team and the nursing team, or even between the Chiefs. It’s all runner talk” (Nurse)*], which is reflected in a diminished knowledge of each patient in the absence of the doctor responsible for him or her. This, in turn, leads to a lower level of learning from the residents, and to sub-optimal solutions to patient-specific issues. Other process losses identified include the frequent unavailability of the patients’ processes where they should be, which was observed by the researchers, and also mentioned by the nurses. It is frequent to have doctors and residents keeping the patients’ files for scientific/academic or learning purposes, and it poses a problem to nurses because then “*we do not have the files, we cannot write down [information] and then there are sheets that remain unfilled.*” Nurses also complain about having multiple, simultaneously, and different requests from several doctors, to which is difficult to give a timely answer. Finally, the four teams do not have a balanced distribution of patients. This happens mostly because one of the senior doctors’ team “*has a lot of patients because their internments are long, because she likes to see everything in detail and takes a long time to discharge them*.” The distribution of patients among the nurses does not account for a qualitative evaluation of the workload “*if I have a patient with high needs plus another 7 or 8, I will just provide the basic care to these, such as giving them their medication, changing diapers and some positioning*.”

At a more micro-level, participants feel the need to work on the emotional part of their job. They consider that “*To deal with death and pain is critical; me and other people, we think that we should talk about it and it is not very well accepted*” (Nurse), and also that they have “*situations of hidden burnout, we try to give those people less unstable patients, but it’s something we really do not talk about*” (Doctor). Furthermore, some participants highlighted that the hardships of dealing with death and suffering become heavier because it is a process that occurs privately [“*We’ve tried to promote the sharing but we cannot because most professionals do not want to talk about it*” (Nurse)], also because of the difficulty to deal with the failure of losing a patient. Particularly from the group of nurses, there is a feeling of low motivation from the team as a whole [“*Difficulty to accept requests for extra work from the Chief, because there is no motivation*” (Nurse)].

To sum up, the Sector faces some constraints related to the ratio of patients (and residents) and available space. This negatively influences their perception of the interpersonal quality of their assistance to the patients. The stress caused by dealing with death and suffering, as well as some process losses also characterize the difficulties reported by the professionals of this sector, while not endangering its technical quality.

### Phase 2: Action Planning

Following the previous data systematization, the project team defined three critical areas of intervention, each with specific broad suggestions: work organization, teamwork, and well-being. The research team shared this list in a first meeting with both the Chief Nurse and the Chief of Sector. The intent of this meeting was to assess whether the perceptions and conclusions drawn from the interviews and observation echoed these professionals’ views, and to define the priorities for intervention. We aimed at starting to define concrete actions or changes together with these two key actors, and to broaden the discussion to the other health professionals afterwards.

The team proposed to discuss how to optimize the management of the professionals, particularly when the occupancy rate was high. This would include to rethink how the patients are attributed to each team (including the qualitative assessment of the patient’s condition) and to each nurse. Still in the work organization area, we also suggested to regulate the temporal duration of the care activities, to define mechanisms to rend the discharge of the patients more expedite, and to create rules for using the patient files. Finally, minimizing the impact of the flow of people in the corridors during the day.

In what teamwork is concerned, our focus was on promoting the inclusion and participation of the ancilliary personnel, and on increasing information sharing. We also advocated an explicit intervention on the well-being and stress levels of professionals, namely through the creation of a space and time to speak about the emotional aspects of the care activity, the contact with patients, and with their families.

### Phase 3: Implementation

Although the Chief Nurse was sympathetic toward our proposal, the Chief of Sector, who, up until this moment had also been supportive of the objectives of the AR, rejected our view on the issues that were relevant to the effective functioning of the sector. In his point of view, those were not the key problems, and he valued more task-related aspects, such as the more complete filing of patient’s records. The emotional dimension of the care and the need of organizational changes, in his opinion, were not so relevant, too. This moment in the project was a strong obstacle, and without the involvement of the Chief Sector, supporting the intervention plan or even contributing with some other ideas to tackling the identified sources of problems, we were unable to move to the third phase, action implementation.

## Discussion

The results from this partial AR project allow for a reflection at different levels: theoretical, methodological, and contextual.

Theoretically, we find the dimensions mentioned by the participants as key obstacles to their performance in Weaver et al. (2013) integrated model for team effectiveness for patient safety in healthcare. The authors propose intra-team processes as a central part of their comprehensive model, including communication, coordination, cooperation, coaching, and adaptation. Communication includes effective strategies for information sharing, highlighting when and how to give and ask for information both in routine contexts, and when facing unpredictable events. Coordination is related to the orchestrating the sequence of necessary actions clearly, namely in the situations where there is the need to reorganize and redefine roles and strategies rapidly, such as when a patient status becomes more critical than what was expected. Cooperation entails considering the needs of others, internal to the team or service, and from other specialties or services, which in turn will reduce information losses and conflict. Coaching, or leadership, intends to develop the autonomy of the team. This happens, for example, when promoting a shared understanding of the clinical situation and of the therapy, as well as by conducting the debriefings with a focus on an improvement of the processes. Finally, adaptation, relates to “adjustments to relevant team processes (i.e., action, interpersonal, and transition) in response to the disruption or trigger giving rise to the need for adaptation” (Maynard et al., 2015, p. 656). Restructuring how the team functions, changing the composition of the team or its resources can be examples of adaptive behavior.

According to the participants in our study, many of these areas were in need of attention. Communication losses were a problem and, more specifically, the inability to discuss those losses. For example, nurses and doctors were unable to discuss and define a strategy for keeping the patient files available for all when necessary. In this particular sector, the nurses’ team was composed of young individuals, and the doctors were either senior specialists or highly competitive residents with an intense focus on academic research. It is likely that real or perceived status differences influenced the communication between the two groups. Fried and Carpenter (2013) relate a similar case among medical records of staff, in which team members voiced their concerns over the preventive services chart plan in private but were reluctant to participate actively in a public discussion. When teams have a high degree of psychological safety, team members may engage in interpersonal risk-taking behaviors, as they feel a “sense of confidence that the team will not embarrass, reject, or punish someone for speaking up” (Edmondson, 1999, p. 354). When team members do not express their needs explicitly to others because they fear the interpersonal consequences of doing so, the potential for conflicts increases. Also, cooperation may diminish, because the needs of others are less valued than the needs of the self. This is clearly seen in the way that the ancilliary personal is “invisible” within the service, with the consequent feelings of low appreciation. Both these situations relate to the core concerns in emotional reactions to conflict, as defined by Fisher and Shapiro (2005), such appreciation and status. Appreciation, the need to feel recognized and to perceive gratitude from others was not fulfilled for the ancilliary personnel, and for the nurses in some circumstances (for example, when receiving multiple requests). Both these professionals also may feel that their input is ignored or less valued by their higher-status counterparts, which can fuel divisions between groups. Indeed, the culture of the sector had a few artifacts (Schein, 1990), or visible symbols, that distinguished between higher and lower status individuals: senior doctors in the service always wear a long dark coat, even if the temperature in the service was high, and the residents would walk behind them.

Despite we formally considered the service as a team, after the data collection, we have strong reasons to question whether they do consider themselves as a team or if they work in the same service, for the same patients but without perceiving all of the professionals as a team. Teams are defined as having common objectives, wide spreading information sharing, cooperation, and particular routines that makes the team unique and different from other teams (e.g., Kozlowski and Bell, 2013). In fact, what we learned from the observation and interviews is that they never refer to the service as a team, using this concept to mention their particular professional groups (medical team, nursing team, etc.). Also, the fact that they do not have well-defined moments to share information, to talk about the patients, the service, and their own work and interaction is another evidence to questioning their team statute.

Another question that should be addressed in this type of interventions, and that was clearly present in our project, is the different status of team members and, in this particular case, between the professional groups. We observed that the professional group more involved in the intervention was the nursing team, the more detached group was the ancilliary personal, but the more powerful group was the medical one, with a very specific hierarchy inside that goes beyond the formal dimension. This can be another obstacle to the AR, stressing the importance of mapping these relationships since the beginning and identifying the risk factors for the intervention. Due to the AR egalitarian nature and its focus on communities priorities (Whitehead et al., 2003), it seems to be crucial to assure that the field is really permeable to this type of intervention and that an empowerment-based approach will not be interpreted as a threat for some groups or members or contributes to perpetuate dysfunctional relations between groups (letting some of these groups outside the process or giving too much power to the dominant groups).

Methodologically, the abrupt ending of the AR due to a blockage from the Chief of sector led to a deep reflection from the research team about the process itself, regardless of the contents and findings. This reflection focused on the role of the Chief of sector. From the initial meeting with him, the team believed that he was “on board,” because he allowed us to conduct the interviews and observations, and was sympathetic toward the idea of having the research team members watching his interactions with both patients and residents. However, it became clear that we should have had extra care in involving him in an *a priori* diagnostic of the sector’s main problems and possible solutions. Another possible methodological change would have been to include him in drafting of the key areas for intervention together with the team, following a presentation of the main results from the observation and interviews. Next, we might have put him in charge of communicating those key areas to the other professionals, keeping his status. According to Salas and Rosen (2013), the changes desired should be embodied by leaders, constantly reinforcing values consistent with teamwork. This particular leader was not aligned with the proposed changes and, as a consequence, the change could not happen within the sector as he was unwilling to move forward with them. We also believe that we could have presented our ideas in a less threatening way for him. Being confronted with a list of topics to be improved could have presented an ego-threat for him, as the Chief of sector.

Regarding this misalignment with the Chief, research has already emphasized the importance of leadership for the intervention success, in supporting organizational occupational health interventions and in the implementation of action plans (e.g., Nielsen, 2017). Due to this, several examples can be found in the literature of how leaders can “make” or “break” an intervention. Leaders may “break it” when not supporting the required changes or being unavailable. Moreover, leaders may intentionally break the intervention. Organizational interventions frequently imply changes to the way work is organized, designed, and managed. New goals may be introduced, roles are changed, and relationship between leaders and workers are renegotiated. Leaders may not be able to make sense of the goals of the intervention or may consider that the intervention is not needed or useful. Nielsen (2017) also emphasize that leaders may resist to a change because they do not feel they have the necessary resources (or even feel to be incompetent) to deal with the changes introduced by the intervention.

Finally, the healthcare context, in itself, can be a barrier to organizational change linked to teamwork. Rosen and Pronovost (2013) advance some specific obstacles that we believe help us frame our experience. According to the authors, communication and teamwork failures are invisible, and only become relevant when they result in critical medical errors with consequences for the patients. This sector, described as “very good technically,” did not have a history of error. On the contrary, all professionals were happy to acknowledge the high quality of care provided to the patients, from an objective point of view – the cure of their clinical conditions or the improvement of their health. Therefore, even if communication between professionals or the emotional environment of the sector were detrimental to their well-being, the fact that the link between those issues and the quality of the service was not clear could have contributed to a negative view on our proposal. Healthcare professionals’ education and training is often rooted on the idea of a “heroic provider” with great technical skills, and the competences for teamwork are not regulated nor incentivized. These social expectations can also impact the ability of health providers and especially of those who are, indeed, considered as excellent providers, to work cooperatively with others.

Following these considerations, it is important to stress some particular conclusions: (i) organizational and team-level constraints in health contexts create specific stress-inducing situations for health professionals that compromise their well-being and put them at higher risk for burnout; (ii) direct hierarchy has a critical role in facilitating (or obstructing) organizational change and the consequent positive (or negative) spirals; (iii) although action-research methodology is an interesting methodology that allows working with people rather than over people, it is very important to ensure some critical conditions as the appropriate involvement with the leadership, assessing the intergroup dynamics and the power distribution inside the team or service, and the alignment between the research team and the participants, building a shared vision of the problems to be solved. Therefore, attempting to intervene directly with the health professionals requires extra care in engaging the leadership and in making sure that their intentions are aligned with the degree of change necessary to improve, not only the quality of the care, but also the well-being of professionals.

Besides the classical three-level of intervention – individual level (e.g., promoting well-being among health professionals through stress manage training), team level (e.g., focusing communication and interpersonal relations), and organizational level (e.g., changing work organization, shifts, or redistributing tasks) – it is also important to provide personal resources to leaders, for instance giving training. This reflection will guide future attempts to intervene in health care services, because training is not always enough. In fact, the complexity of health environments/contexts might support or undermine the training transference for the real situation.

These arguments emphasize the critical role of the first stage of intervention. Besides assessing the needs, the diagnose should cover the “assessment” of leader’s readiness for change and determine if it is the right time and if there are conditions to introduce the intervention. Before starting an organizational occupational health intervention, we need to consider the shared perceptions of leaders and team members about the working climate and take additional initiatives to correct any misalignments, in order to prevent risks for the project and making possible to intervene in healthcare contexts.

## Data Availability Statement

The raw data supporting the conclusions of this article will be made available by the authors, without undue reservation.

## Ethics Statement

The studies involving human participants were reviewed and approved by Comissão de Ética para a Saúde, Hospital de Santa Maria, CHLN. The patients/participants provided their written informed consent to participate in this study.

## Author Contributions

All authors contributed to conception and design of the study. SR and PC collected the data. SR, PC, and AP analyzed the data and organized the database. SR, PC, AP, and SS wrote sections of the manuscript. All authors contributed to manuscript revision, read, and approved the submitted version.

### Conflict of Interest

The authors declare that the research was conducted in the absence of any commercial or financial relationships that could be construed as a potential conflict of interest.
